# Association between pain threshold and manifested pain assessed using a PD-specific pain scale in Parkinson's disease

**DOI:** 10.3389/fneur.2024.1420696

**Published:** 2024-07-25

**Authors:** Galina Stoyanova-Piroth, Ivan Milanov, Katerina Stambolieva

**Affiliations:** ^1^St. Naum Hospital of Neurology and Psychiatry, Medical University, Sofia, Bulgaria; ^2^ZURZACH Care, Neurorehabilitation, Rehaklinik Baden Dättwil, Baden, Switzerland; ^3^Institute of Neurobiology, Bulgarian Academy of Sciences, Sofia, Bulgaria

**Keywords:** Parkinson's disease, pain threshold, nociceptive reflex, manifested pain, KPPS

## Abstract

**Background:**

The neurodegenerative process in Parkinson's disease (PD) affects both dopaminergic and non-dopaminergic structures, which determine the wide range of motor and non-motor symptoms (NMS), including different types of pain. Diverse mechanisms contribute to pain in PD. Abnormal nociceptive processing is considered a distinctive feature of the disease.

**Objective:**

In the present study, we used a validated PD-specific pain assessment tool to investigate self-reported pain in PD patients and to analyze the association with the objective pain threshold.

**Methods:**

The RIII component of the nociceptive flexor reflex was assessed in 35 patients with PD and was compared to 40 healthy controls. Self-reported pain was measured using the Bulgarian version of the King's Parkinson's Disease Pain Scale (KPPS-BG). A correlation analysis was used to investigate the relationship between the objective nociceptive threshold and PD pain as assessed by KPPS-BG.

**Results:**

PD patients had a significantly lower RIII threshold than control individuals (the mean SD value was 6.24 ± 1.39 vs. 10.33 ± 1.64) when assessed in the “off” state. A statistically significant (*p* < 0.05) fairly negative Spearman's correlation was observed between the decreased spinal nociceptive threshold and fluctuation-related pain (−0.31). Domain 4, “nocturnal pain” (−0.21), and the KPPS-BG total score (−0.21) showed a weak negative correlation. An insignificant positive correlation was found between domain 6—“discoloration, edema/swelling”—and the RIII threshold. A higher Movement Disorders Society Unified Parkinson's Disease Rating Scale (MDS-UPDRS) part III score and modified Hoehn and Yahr (H&Y) scale are associated with a decreased nociceptive flexor reflex threshold.

**Conclusion:**

The results of the present study demonstrate the important role of increased spinal nociception in the occurrence of pain, which is associated with fluctuations and, to a lesser extent, nocturnal pain.

## Introduction

Parkinson's disease (PD) is the second most common neurodegenerative disease (1) and the most common neurodegenerative movement disorder (2). In comparison to other neurological diseases, PD has the fastest-growing prevalence, disability, and mortality (3). The cardinal symptoms of the disease, including tremors, bradykinesia, rigidity, and postural instability, result from the degeneration of dopaminergic neurons in the substantia nigra pars compacta and reduced dopamine levels in the nigrostriatal pathways (4). The neurodegenerative process also involves cholinergic, noradrenergic, and serotonergic structures, resulting in a wide range of non-motor symptoms (NMS), (5) such as autonomic, neuropsychiatric, sleep, and sensory disturbances, including pain (6).

The etiology of pain in Parkinson's disease (PD) is multifactorial (7). The progressive degeneration characteristic of the disease is multifocal and can affect pain processing at multiple levels (8). This altered pain processing in the brain and the spinal cord is likely related to dopaminergic mechanisms. However, the involvement of non-dopaminergic structures is also assumed (7, 9).

The diversity of mechanisms contributing to pain is reflected by heterogeneous pain phenotypes. As a result, several attempts have been made to classify pain in PD, for example, according to motor status, pain dimensions, and subtypes. The most recent classification is based on mechanistic descriptors (10).

As the summation of the afferent somatosensory nociceptive inputs occurs in the spinal cord and is followed by the activation of the efferent motor neuron, the abnormal nociceptive input processing in PD may lead to the facilitation of the nociceptive flexion reflex (NFR). An additional factor can be the diminished inhibitory control from descending pain pathways (11).

The flexor reflex combines the plantar reflex described by Wernicke in 1881 and the dorsal flexion of the foot with flexion at the knee and hip joints (12). This protective spinal exteroceptive reflex of the lower limbs is intended to withdraw the limb from the irritating stimulus (13). Kugelberg was the first to investigate the electromyographic characteristics of the flexor reflex in 1948 (12). The reflex response consists of the RII (tactile) and RIII components. The RIII component is nociceptive and correlates with the pain threshold (14).

The pain threshold evaluates the sensory-discriminative aspect of pain (15). An anatomical substrate of this component is the lateral nociceptive system (16).

A lower pain threshold could be detected in patients with PD, which probably, in combination with additional factors, leads to pain manifestation (9). The results of meta-analyses demonstrate that hyperalgesia contributes to clinical pain in PD patients (17). Using a quantitative pain assessment method, a recent study by Kurihara et al. found a positive correlation between current perception threshold and disease duration and stage in PD patients with pain. According to the authors, these results reflect a peripheral neuropathy developing with the disease progression (18).

However, the contribution of nociceptive hypersensitivity to the development of PD-specific spontaneous pain is not fully understood. The objective pain threshold using NFR has long been demonstrated to be reduced in PD patients with (19) and without clinically expressed pain (20, 21). Reduced pain threshold and pain tolerance in PD patients with dystonic and non-dystonic pain (including musculoskeletal pain and central and peripheral neuropathic pain) and pain-free PD patients were published (22).

Although several instruments have been developed to measure pain in the general population, the clinical presentation and etiology of pain in PD are heterogeneous (7). This partly explains the difficulty in developing formal consensus-based guidelines for assessing and managing pain in PD. The King's Parkinson's Pain Scale (KPPS) is the first standardized scale developed by Chaudhuri et al. in 2015 to categorize PD pain levels in terms of severity and frequency into seven different domains: (1) musculoskeletal pain; (2) chronic pain; (3) fluctuation-related pain; (4) nocturnal pain; (5) oro-facial pain; (6) discoloration, edema/swelling; and (7) radicular pain (23).

The present study aims to investigate the relationship between the objective nociceptive threshold and spontaneous PD pain evaluated by the Bulgarian version of the KPPS.

## Materials and methods

### Participants

A total of 35 consecutively enrolled patients with PD based on the UK Parkinson's Disease Society Brain Bank criteria (24) and 40 healthy controls participated in the study. The patients were recruited from the Movement Disorders Department of the University Hospital of Neurology and Psychiatry “St. Naum,” Sofia, Bulgaria. Patients with cognitive impairment, who were tested with a Mini-Mental State Examination (score < 24), other chronic pain conditions, and atypical or secondary Parkinsonism were excluded.

The PD progression stage was assessed using the modified Hoehn and Yahr (H&Y) scale (25). The motor evaluation was performed using the Movement Disorders Society Unified Parkinson's Disease Rating Scale (MDS-UPDRS) part III (26), and the motor subtype was determined (27).

All patients and controls provided written informed consent before participating in the study. The study was approved by the local ethics committee and was conducted in accordance with the ethical standards outlined in the Declaration of Helsinki.

### Subjective pain assessment

The Bulgarian versions (BG) of the King's Parkinson's Disease Pain Questionnaire (KPPQ) and King's Parkinson's Disease Pain Scale (KPPS) were used to examine the clinical expression of pain. KPPS-BG has the same construction as the original scale (28).

The Numerical Rating Scale (NRS) was used for a subjective evaluation of the pain intensity. Participants were instructed to rate the pain intensity of each stimulus, ranging from 0 (“no pain”) to 10 (“the worst pain imaginable”).

### Objective pain assessment

To study NFR, we used Willer's method by stimulating the n. suralis with a bipolar surface electrode in the region of the lateral malleolus and eliciting a reflex response from the short head of the m. biceps femoris with an inter-electrode distance of 3 cm. The participants were examined in a quiet room with an optimal air temperature of 20–22°C, in a supine position with complete muscle relaxation. Before the placement of the electrodes, the skin in the lateral retromalleolar region and over the short head of the biceps femoris muscle (5 cm proximal to the popliteal fossa in the lateral posterior femoral surface) was treated with 70% ethyl alcohol and a fine exfoliating gel. A series of five rectangular electric pulses with a frequency of 200 Hz, a duration of each individual pulse of 1 ms, and a duration of the entire series of 50 ms was used. The threshold of the RIII reflex was determined by the intensity of electrical stimulation using the “staircase (up-down)” method. An average of three appearance and three disappearance values of the reflex response corresponding to the pain threshold was calculated (14). All patients were examined in an off medication state (at least 12 h after the last administration of the dopaminergic medication).

### Statistical analysis

Data are presented as mean ± standard deviation. All statistical analyses were conducted after corresponding assumption checks for normality of distribution using the Shapiro–Wilk test. The Mann–Whitney *U*-test was applied to compare measurements between patients and healthy controls, and the Wilcoxon rank was used to compare measurements on both sides of the body. Spearman's correlation was applied to evaluate the correlation between the RIII threshold and patients' clinical characteristics, KPPQ-BG, and KPPS-BG scores. The strength of the correlation was determined as follows: correlation coefficients between 0.1 and 0.2 indicate a weak correlation, coefficients between 0.3 and 0.5 indicate a fair correlation, coefficients between 0.6 and 0.7 indicate a moderate correlation, and coefficients between 0.8 and 1 indicate a strong correlation (29, 30). The significance level was set at a *p*-value of < 0.05. Statistical analyses were performed using the computer software Statistica 8.0 for Windows (Stat Soft Inc. USA).

## Results

A total of 35 patients (21 men and 14 women) and 40 healthy controls (21 men and 19 women) participated in this study. The mean age of the patients was 62.5 ± 7.9 years (median 62.0), and the mean age of the controls was 61.7 ± 7.5 years (median 62.0). Among the patients, 34% were drug-naive, 57% had a levodopa equivalent daily dose (LEDD) of < 1,000 mg, and 9% had an LEDD of >1,000 mg. The clinical data of all patients with PD are presented in Table 1. The electrophysiological measurements were performed in the off state.

**Table 1:** Clinical characteristics of patients with Parkinson‘s disease (*n* = 35).

**Patients with PD**		
**Characteristics**	**Mean**	**SD**
Duration of PD, years	3.9	2.8
Age at onset, years	58.7	9.1
Modified H&Y scale	2.1	0.7
MDS-UPDRS III	24.63	10.8
MMSE	28.86	1.38
BDI	4.81	2.82
KPPS-BG total score	18.31	12.4
Domain 1: Musculoskeletal pain	4.71	3.6
Domain 2: Chronic pain	1.54	2.9
Domain 3: Fluctuation-related pain	3.68	4.5
Domain 4: Nocturnal pain	4.91	5.4
Domain 5: Oro-facial pain	1.12	3.1
Domain 6: Discoloration, edema/swelling	0.71	1.85
Domain 7: Radicular pain	1.62	2.5

PD, Parkinson's disease; SD, Standard deviation; H&Y scale, Hoehn and Yahr scale; MDS-UPDRS III, Movement Disorder Society Unified Parkinson's Disease Rating Scale-part III; MMSE, Mini-Mental State Examination; BDI, Beck Depression Inventory; KPPS-BG, Bulgarian version of the King's Parkinson's Disease Pain Scale.

A fair correlation between the RIII threshold and the MDS-UPDRS III (Spearman‘s Correlation −0.37, *p* < 0.05) and between the RIII threshold and the modified H&Y scale (Spearman‘s Correlation −0.35, *p* < 0.05) was observed.

The pain intensity reported by patients and healthy subjects using NRS was similar (for patients, the mean value was 4.03 ± 1.61, and for healthy, the mean value was−4.01 ± 1.36, using the Mann–Whitney *U*-test, with *p* > 0.05).

A significant difference in the RIII threshold between both sides of the body (left and right) for healthy controls was not established. The patients' RIII threshold for the more affected right or left side was not statistically significant. The RIII threshold of the dominant side for the disease was significantly lower than that of the contralateral body side (Wilcoxon matched pairs test, *p* < 0.001). The RIII threshold for both sides in patients was found to be lower than that observed in healthy subjects. However, a significant difference in the RIII threshold was only observed for the more affected side (mean ± SD was 6.24 ± 1.39 vs. 10.33 ± 1.64) compared to healthy subjects (Mann–Whitney *U*-test, *p* < 0.001; Table 2).

**Table 2:** The RIII thresholds by patients (dominant for the disease and less affected body side) and controls.

**RIII threshold**	**Body side**	**Number**	**Median**	**IQR, (25th,75th percentiles)**
Patients (in/off)	More affected side	35	6.0^*∧^	1.7 (5.1, 6.8)
	Less affected side	35	6.8	2.1 (5.8, 7.9)
Healthy subjects	Both sides	80	10.2	2.2 (8.9, 11.1)
	Left side	40	10.1	2.5 (8.9, 11.4)
	Right side	40	10.2	2.4 (8.9, 11.3)

IQR, interquartile range (25th, 75th percentiles).

^*^Significant difference between patients and healthy subjects on both sides (Mann–Whitney U-test, p < 0.001).

^∧^Between the more and less affected side in patients (Wilcoxon matched pairs test, p < 0.001).

Table 3 presents Spearman's correlations between all items in KPPQ-BG and the RIII threshold of the more affected body side. A fair negative correlation was found between item 4 and the RIII threshold. A weak negative correlation was found for the other four items: 5, 6, 9, and 14. For items 2 and 3, a positive correlation was observed.

**Table 3:** Spearman's correlations between the RIII thresholds in the off state of the dominant side and items in the KPPQ-BG for patients with PD (*n* = 35).

**KPPQ-BG items vs. RIII-affected side**	**Spearman's correlations (rs) *p* (2-tailed)**
1. Pain around joints (musculoskeletal)	0.02 (*p* = 0.91)
2. Pain related to internal organs	0.28 (*p* = 0.098)
3. Generalized non-specific pain in the stomach area	0.30 (*p* = 0.072)
4. Pain deep within the body	−0.37 (*p* = 0.030)
5. Dyskinetic pain	−0.27 (*p* = 0.13)
6. Painful muscle cramps in a specific area during the “off” period	−0.20 (*p* = 0.23)
7. Generalized “off” period pain	−0.07 (*p* = 0.67)
8. PLM or RLS-associated pain	−0.09 (*p* = 0.61)
9. Pain while turning in bed at night	−0.16 (*p* = 0.33)
10. Pain when chewing	−0.07 (*p* = 0.67)
11. Pain due to grinding teeth during the night	−0.09 (*p* = 0.61)
12. Burning sensation in the mouth	−0.08 (*p* = 0.65)
13. Burning pain in the limbs	−0.08 (*p* = 0.65)
14. Shooting pain/pins and needles down the limbs	−0.12 (*p* = 0.49)

KPPQ-BG, Bulgarian version of the King's Parkinson's Disease Pain Questionnaire.

A significantly negative fair correlation was found between the RIII threshold of the more affected side and domain 3, “fluctuation-related pain.” Domain 4, “nocturnal pain,” and the KPPS-BG total score demonstrated a weak negative correlation, while domain 6 exhibited a weak positive correlation with the RIII threshold (Table 4).

**Table 4:** Spearman's correlations between the RIII thresholds (in the off state) of the dominant side and domains and total score of KPPS-BG for patients with PD (*n* = 35).

**KPPS-BG scores vs. RIII affected side**	**Spearman's correlations (rs) *p* (2-tailed)**
Domain 1: Musculoskeletal pain	−0.017 (*p* = 0.34)
Domain 2: Chronic pain	−0.025 (*p* = 0.88)
Domain 3: Fluctuation-related pain	−0.31 (*p* = 0.029)
Domain 4: Nocturnal pain	−0.21 (*p* = 0.22)
Domain 5: Oro-facial pain	−0.09 (*p* = 0.61)
Domain 6: Discoloration, edema/swelling	0.12 (*p* = 0.49)
Domain 7: Radicular pain	0.07 (*p* = 0.67)
Total score	−0.21 (*p* = 0.28)

KPPS-BG, Bulgarian version of the King's Parkinson's Disease Pain Scale.

## Discussion

In accordance with previous data (19, 20), the present study reveals a lower RIII threshold in the cohort of 35 PD patients when examined in an off state compared to the healthy controls. Although the RIII threshold is decreased for both sides of the body in PD patients compared to healthy subjects, a statistically significant difference was found for the more affected side (Table 2). This altered spinal nociception is associated with a higher MDS-UPDRS III score and a modified H&Y stage.

We found a negative correlation between the reduced electrical pain threshold and domain 3, fluctuation-related, and domain 4, nocturnal pain, and a negligible positive correlation with domain 6—discoloration, edema/swelling—. There was no association found between spinal hyperalgesia and musculoskeletal, chronic, oro-facial, or radicular pain.

A number of studies have shown that musculoskeletal pain is the most common type of pain (31, 32) reported by patients with PD. Using a different study protocol, Tinazzi et al. suggested that muscular pain in PD patients results from abnormal processing of nociceptive inputs (33). The lack of correlation between the reduced nociceptive threshold and domain 1 in our study suggests that impaired nociception is insufficient for the clinical manifestation of this type of pain. A combination of hyperalgesia and other factors is probably important.

Risk factors for pain have been recognized for the female sex (34), genetic factors (35), the age of onset (36), motor (rigidity, stiffness, and diminished mobility) (37, 38) and non-motor (depression, anxiety) (39) manifestations of the disease, autonomic symptoms (32), and medical disturbances associated with painful conditions (22). An association was found between the female sex and L-dopa equivalent daily dose (LEDD) and musculoskeletal pain in PD patients. However, the study did not reveal an apparent relationship between musculoskeletal pain and motor symptoms. The authors included only patients with pain which improved after levodopa or mobility (40).

A *post-hoc* analysis revealed a statistically significant (*p* < 0.05) negative correlation between item 4, “Pain deep within the body,” and a positive correlation between item 2, “Pain related to internal organ,” and the objective pain threshold. Both questions constitute domain 2, “Chronic pain.” The recently established Parkinson's Disease Pain Classification System categorizes chronic pain related to Parkinson's as nociceptive, neuropathic, or nociplastic (41). Abnormal cortical processing of nociceptive inputs was earlier suggested as a pathophysiological mechanism for developing chronic pain in PD (9). Recently, abnormal pain-motor integration was shown to be involved in the mechanism of chronic pain in PD (42). Although chronic visceral pain is also characterized by hypersensitivity and hyperalgesia, differences exist in the pathophysiological mechanisms between visceral and somatic chronic pain (43).

Moreover, in PD patients, visceral pain shows a weak association with constipation scores and autonomic symptoms (32). Our data suggest that patients with a tendency toward a normal or higher threshold develop more often pain related to internal organs. Since the two items are mutually exclusive, showing positive and negative correlations with the pain threshold, the domain demonstrated no correlation. A cross-sectional study published in 2023 revealed a distinct somatosensory and cortical neurophysiological profile between patients with different types of chronic pain. Barboza et al. found that patients with nociceptive pain have a lower detection threshold for warm and mechanical stimuli and a lower rest motor threshold when compared with patients with non-nociceptive pain. The patients were examined in the on state (44).

A statistically significant fairly negative Spearman's correlation was observed between the decreased spinal nociceptive threshold and domain 3, “fluctuation-related pain.” This domain combines off-dystonic and dyskinetic pain. Dyskinesia and motor fluctuations are the primary complications of L-dopa therapy (45). Their frequency and severity are the same regardless of the duration of levodopa treatment (46). However, the severity of the disease and the L-dopa dose are more important in the development of dyskinesia in PD (47). Motor complications (e.g., dyskinesia) are recognized as one of the predictors of PD pain (48). Conversely, PD patients who report pain more often suffer from motor fluctuations (49, 50). Patients with dyskinesia have increased pain sensitivity (51). A common pathophysiological mechanism has been suggested between dyskinesia and pain in PD (52). Recently, Sung et al., in a functional imaging study, confirmed the increased pain sensitivity in PD patients with dyskinesia. This observation could be explained by the occurrence of central sensitization of pain pathways in dyskinetic PD patients (51). Both dopaminergic and non-dopaminergic mechanisms have been discussed as potential contributors. It is also hypothesized that changes in descending pain inhibition may lead to increased pain sensitivity, as observed in dyskinetic PD patients (51).

Based on our results, we can hypothesize that in PD patients with fluctuation-related pain, in addition to the primary hyperalgesia, which is induced by dopaminergic (17) and probably non-dopaminergic (9) mechanisms, plastic changes due to the pulsed dopaminergic stimulation in motor (striatum) and non-motor pathways (53) are superimposed.

A weak negative correlation was observed for domain 4, “nocturnal pain,” which expresses the correlation between experiencing night-time pain while turning in bed and a reduced pain threshold. This pain most likely reflects nocturnal akinesia (23). This type of relationship between pain, severity of motor symptoms, and longer off-state duration (50) was also found. Nocturnal akinesia can lead to sleep disruption (54), which, in turn, can increase pain sensitivity and vulnerability to pain (55).

Domain 5—“oro-facial pain” contains questions related to 10, “pain when chewing;” 11 “pain due to grinding teeth during the night;” and 12, “burning sensation in the mouth.” None of these pain types showed an association with hyperalgesia. A recent systematic review reveals that orofacial pain in PD patients is more prevalent than in controls. Furthermore, this prevalence will be higher when the disease severity gets worse (56). Risk factors for the development of orofacial pain in PD are the pathology of the trigeminal nerve (57), restrictions in movements (58), and swallowing and speech problems (59). Pain when chewing is a result of temporomandibular joint disorders (TMD) (60) and patients with PD have more difficulties when chewing compared to healthy controls (56). Recently, a pilot study revealed a significant relationship between sleep and awake bruxism and PD, as well as between orofacial pain, possible TMD pain, and PD (58). In patients with a burning sensation in the mouth, a cobalamin deficiency should be excluded, especially with the advance of the disease (61).

We also found a weak positive correlation between the item “generalized non-specific pain in the stomach area,” and domain 6—“discoloration, edema/swelling.” The second item that composes this domain, “burning pain in the limbs,” expresses neuropathic pain and shows no correlation with the lowered threshold. Thus, the statistical analysis revealed a negligible positive correlation for domain 6.

In addition to the increased pain sensitivity, other peripheral factors, such as postural changes and bone abnormalities observed in PD, may exacerbate or even cause radicular pain (62).

Our study found a correlation between disease severity (the modified H&Y scale and the MDS-UPDRS III score) and the reduced pain threshold. These results are in concordance with previous data (22), showing that the severity of motor symptoms is correlated with both decreased sensory thresholds and spontaneous pain. Recently, a study revealed the relationship between a current perception threshold and the disease duration and severity in PD patients with pain. The authors did not provide a correlation analysis between the pain threshold and PD-specific pain types (18). Using a different methodology, the current study describes a specific relationship between objective nociceptive threshold and spontaneous PD pain, which may prove beneficial in the clinical setting.

In summary, the obtained results show that increased pain sensitivity is an intrinsic factor in PD but is not sufficient to explain the clinical manifestation of all PD-specific pain.

The present study has some limitations: the patient population does not equally represent all H&Y stages of the disease. Although the RIII measures the pain threshold and expresses the sensory-discriminative component of pain, no data are available regarding the emotional-motivational aspect, which could be probably predominantly impaired in patients with PD (63), or on the cognitive-evaluative aspect. Future studies investigating all aspects of nociception in each type of PD pain are warranted.

## Data availability statement

The original contributions presented in the study are included in the article/supplementary material, further inquiries can be directed to the corresponding author.

## Ethics statement

The studies involving humans were approved by Ethics Committee of the University Hospital for Active Treatment in Neurology and Psychiatry “St. Naum” Sofia, Bulgaria. The studies were conducted in accordance with the local legislation and institutional requirements. The participants provided their written informed consent to participate in this study.

## Author contributions

GS-P: Conceptualization, Funding acquisition, Investigation, Methodology, Visualization, Writing – original draft, Writing – review & editing. IM: Conceptualization, Funding acquisition, Investigation, Methodology, Project administration, Resources, Supervision, Writing – review & editing. KS: Formal analysis, Methodology, Software, Supervision, Validation, Visualization, Writing – original draft, Writing – review & editing.
